# Low-Density Lipoprotein (LDL)-Antioxidant Biflavonoids from *Garcinia madruno*

**DOI:** 10.3390/molecules18056092

**Published:** 2013-05-22

**Authors:** Edison Osorio, Julián Londoño, Jaume Bastida

**Affiliations:** 1 Grupo de Investigación en Sustancias Bioactivas, Facultad de Química Farmacéutica, Universidad de Antioquia, Carrera 53 n 61-30., A.A. 1226, Medellín, Colombia; E-Mail: jalondo@gmail.com; 2 Departament de Productes Naturals, Facultat de Farmàcia, Universitat de Barcelona, Avn. Joan XXIII s/n, Barcelona 08028, Spain; E-Mail: jaumebastida@gmail.com

**Keywords:** *Garcinia madruno*, Clusiaceae, bioflavonoids, LDL-antioxidant

## Abstract

Six biflavonoids were isolated from *G. madruno*, one of which, 7''-*O*-(6''''-acetyl)-glucoside of morelloflavone, is a new compound identified on the basis of 1D, 2D NMR (HMQC and HMBC) spectroscopic methods and chemical evidence. The antioxidant activity of the biflavonoids against low-density lipoprotein (LDL) peroxidation induced with Cu^2+^, was studied by means of a TBARS assay. The antioxidant potential of a biflavonoid fraction (BF) was also evaluated and correlated with its biflavonoid content. The flavanone-(3→8'')-flavone biflavonoids displayed antioxidant activity, particularly morelloflavone, which was significantly more potent than quercetin, with a CE_50_ of 12.36 μg/mL. Lipid peroxidation, was also significantly reduced in the presence of the BF (EC_50_ = 11.85 μg/mL). These results suggest that the BF is an excellent antioxidant.

## 1. Introduction

*Garcinia madruno* (Kunth) Hammel, commonly known as madroño, is a tree endemic to Central and South America. It is resistant to plagues and illnesses and adaptive to different environmental conditions [1]. Extracts obtained from *G. madruno* show antibacterial activity and are particularly efficient against *Staphylococcus aureus* [2]. Other species of the *Garcinia* genus have been reported to exhibit diverse biological properties, such as anti-inflammatory, antioxidant, antiimmunosuppressive, antitumor promoter, cytotoxicity, antinematodal, antiviral, antiplasmodial, trypanocidal, and antimicrobial activity, and also in healing skin infections and wounds [3,4,5,6,7,8,9,10]. Phytochemical studies of this genus have revealed the presence of xanthones, benzophenones and biflavonoids [3,11,12,13]. Consequently, we became interested in carrying out a comprehensive investigation of the twigs and leaves of *G. madruno*. In a previous investigation, we report the inhibitory LDL oxidation potential and free radical stabilization capacity of a biflavonoid fraction (FB) from *G. madruno* [14]. This paper deals with the isolation and characterization of a new biflavonoid, along with five known biflavonoids. The relative antioxidant activity of the biflavonoids against LDL peroxidation is also reported.

## 2. Results and Discussion

### 2.1. Structure Elucidation

**Figure 1:**
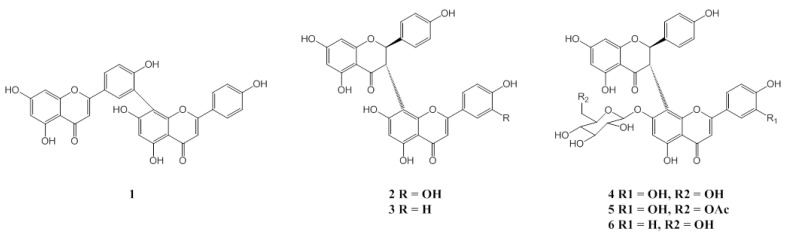
Chemical structures of compounds **1**–**6**.

Extensive column chromatography of EtOAc and MeOH extracts of *G. madruno* produced a new biflavonoid **5**, along with amentoflavone (**1**), morelloflavone (**2**), volkensiflavone (**3**), fukugiside (**4**) and spicataside (**6**) (Figure 1).

Compound **5** was obtained as a brown powder, mp 268–270 °C. In the TLC analyses a dark green spot was seen with ferric chloride, indicating its phenolic nature. In the ^1^H-NMR spectrum, recorded in CD_3_OD at room temperature, major peaks were accompanied by less intense corresponding peaks with close chemical shifts (Table 1). The ^13^C-NMR signals in CD_3_OD at room temperature were equally complex. The doubling of signals in the ^1^H and ^13^C-NMR of **5** suggested the existence of two conformers due its rotational behaviour (atropisomerism) [15,16]. This phenomenon represents a characteristic of flavanone-(C-3→C-8'')-flavone biflavonoids [15,17], which was observed in all isolated compounds of this kind (compounds **2**–**6**). In fact, the *Garcinia* biflavonoids generally leading to C-3→C-8'' biflavanones or C-3→C-8'' flavanone-flavone carry at least one stereogenic center, but also show atropisomeric behavior due to restricted rotation about the central axis [15,18].

**Table 1:** ^1^H-NMR, ^13^C-NMR, and HMBC data of compound **5** in CD_3_OD.

	^1^H *δ* (*J* in Hz)			^13^C		HMBC
	**5a**	**5b**		**5a**		**5a**
2	5.76 *d* (12.0)	5.76 d**		82.87 *d*		C-1'
3	4.81 *	5.33 *d* (12.0)		51.21 *d*		C-1', C-7'', C-9''
4				197.36 *s*		
5				165.84 *s*		
6	5.96 br*s*	5.96 br*s*		99.61 *d*		C-8, C-10
7				164.92 *s*		
8	5.96 br*s*	5.96 br*s*		96.65 *d*		C-10
9				168.51 *s*		
10				103.65 *s*		
1'				130.55 *s*		
2'	7.05 *d* (8.8)	7.11 *d* (8.0)		129.33 *d*		C-2, C-4', C-6'
3'	6.35 *d* (8.4)	6.35 *d* (8.4)		115.51 *d*		C-1', C-5'
4'				158.53 *s*		
5'	6.35 *d* (8.4)	6.35 *d* (8.4)		115.51 *d*		C-1', C-3'
6'	7.05 *d* (8.8)	7.11 *d* (8.0)		129.33 *d*		C-2, C-2', C-4'
2''				166.28 *s*		
3''	6.41 *s*	6.52 *s*		104.49 *d*		C-1'''
4''				184.04 *s*		
5''				162.75 *s*		
6''	6.66 *s*	6.56 *s*		100.01 *d*		C-8'', C-10''
7''				161.62 *s*		
8''				104.94 *s*		
9''				156.80 *s*		
10''				106.74 *s*		
1'''				123.09 *s*		
2'''	7.31 br*s*	7.26 br*s*		114.38 *d*		C-6'''
3'''				146.92 *s*		
4'''				151.35 *s*		
5'''	6.88 *d* (8.4)	6.61 *d* (8.4)		116.97 *d*		C-3'''
6'''	7.27 br*d* (8.0)	7.10 brd**		120.89 *d*		C-2'', C-2''', C-4'''
1''''	5.24 *d* (7.6)	5.17 *d* (8.0)		101.32 *d*		C-7''
2''''	3.34 *m*	3.34 m**		75.24 *d*		
3''''	3.38 *dd* (9.2, 8.8)	3.38 m**		78.22 *d*		
4''''	3.82 *m*	3.82 m**		75.73 *d*		
5''''	3.60 *ddd* (11.2, 6.2, 2.3)	3.60 m**		71.36 *d*		
6''''a	4.27 *dd* (12.0, 2.3)	4.27 m**		64.54 *t*		OCOMe
6''''b	4.13 *dd* (12.0, 6.4)	4.13 m**				
OCOMe				172.75 *s*		
Me	1.95 *s*	2.03 *s*		20.77 *q*		OCOMe

Series a and b represent major and minor conformers at 25 °C, respectively. * Overlapping with solvent signals. ** Not identified due to overlapping.

It is found that the NMR chemical shifts of **5 **were very similar to those of the known compound fukugiside [19], which has 42 mass units less than **5**. The ^1^H and ^13^C-NMR signals of the major conformer **5a** showed two carbonyls at δ_C_ 197.36 (*s*, C-4) and δ_C_ 184.04 (*s*, C-4'') and an additional carbonyl group δ_C_ 172.75 (*s*, OCOCH_3_), compared with the NMR signals of **4**, also an aromatic proton δ_H_ 6.66 (1H, *s*), assigned to C-6'' position due to the long-range correlation with C-8'' and C-10''. By HMBC correlations, two aromatic protons at δ_H_ 5.96 (2H, br*s*) were assigned to be located at C-6 and C-8, and the singlet at δ_H_ 6.41 (1H, *s*) were attributed to the positions C-3''. In addition, resonances for an A_2_B_2_ spin system that is comprised of four protons in two doublets at δ_H_ 7.05 (2H, *d*, *J* = 8.8, H-2'/6') and 6.35 (2H, *d*, *J* = 8.4, H-3'/5') assignable to a 4-subtituted B-ring were also observed. Three other aromatic proton signals at δ_H_ 6.88 (1H, *d*, *J* = 2.0 Hz, H-5'''), 7.27 (1H, *brd*, *J* = 8.0, H-6'''), and 7.31 (1H, *brs*, H-2''') corresponded to an ABX system of E-ring. The HMBC spectrum confirms this system by the long-range correlations of the aromatic proton H-6''' with C-2'' (δ_C_ 166.28, *s*), C-2''' (δ_C_ 114.38, *d*), and C-4''' (δ_C_ 151.35, *s*). For the minor conformer **5b**, was possible only to assign its ^1^H-NMR signals since the ^13^C NMR signals were generally of low intensity.

The ^1^H-NMR spectrum of **5 **showed one anomeric proton at δ_H_ 5.24 (1H, *d*, *J* = 7.6 Hz), and together with the corresponding carbon resonances at δ_C_ 101.32 (*d*, C-1'''') it was easily deduced that compound **5 **contained an *O*-β-d-glucopyranose moiety [20,21]. Other signals to this unit correspond to δ_H_ 3.34 (1H, *m*, H-2''''), 3.38 (1H, *dd*, *J* = 9.2, 8.8, H-3''''), 3.82 (1H, *m*, H-4''''), 3.60 (1H, *ddd*, *J* = 11.2, 6.2, 2.3, H-5''''), 4.27 (1H, *dd*, *J* = 12.0, 2.3, H-6''''a) and 4.13 (1H, *dd*, *J* = 12.0, 6.4, H-6''''b). HMBC correlations of anomeric proton, H-3 (δ_H_ 4.81) and H-6'' with C-7'' (δ_C_ 161.62, *s*) were also observed, which confirmed the link between the two flavonoid moieties via C-3 and C-8'' and the binding of glucose to C-7'' of the aglycone. The ^1^H- and ^13^C-NMR spectra of compound **5** were compared with the data of fukugiside (**4**) and an additional acetyl group with three-proton singlet at δ_H_ 1.95 (3H, *s*, OCOCH_3_) and a carbonyl carbon at δ_C_ 172.75 were observed. In addition, the downfield shift of the ^1^H-NMR signals at H-6'''', along with a downfield shift at C-6'''' (δ_C_ 64.54, *t*), suggests that the acetyl group is attached to the 6-hydroxyl group of glucose [21,22]. The placement of the acetoxy group at C-6'''' was supported by the HMBC spectrum that displayed correlations between H-6''''a and the methyl protons with the carbonyl carbon.

**Figure 2:**
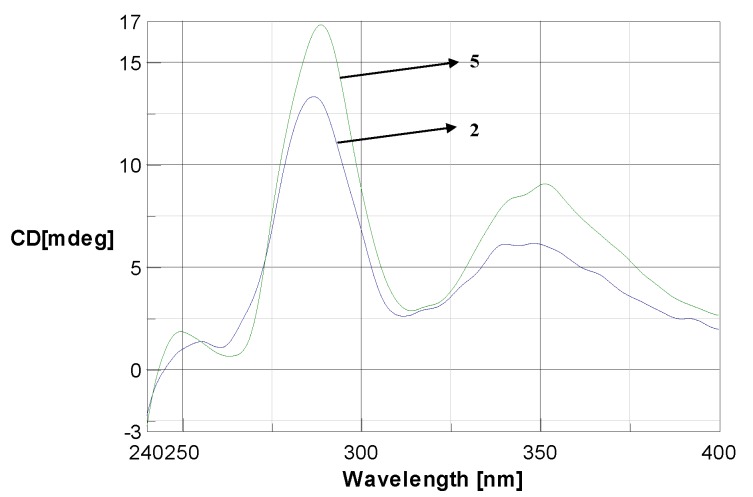
CD spectra of compounds **5** and its aglycone **2**.

The relative stereostructure of **5** between H-2 and H-3 was identified as *trans* by considering the coupling constant of *J*_2,3_ = 12 Hz and the lack of NOE effect between these protons. The absolute stereochemistry was deduced to be 2*R*,3*S* by considering the observed positive optical rotation and CD curves with maximum values of around 351 and 288 nm, qualitatively similar to the CD curve of the morelloflavone (**2**) [15,23] (Figure 2). Based on the spectroscopic data of **5**, the structure was unambiguously assigned as a new biflavonoid, 7''-*O*-(6''''-acetyl)glucoside of morelloflavone, and named madrunoudeaside,. Comparisons of NMR and MS data for the known compounds **1**–**4** and **6** with reported values led to their identification as amentoflavone (**1**) [24,25], morelloflavone (**2**) [15,23], volkensiflavone (**3**) [26,27], fukugiside (**4**) [19], and spicataside (**6**) [28], respectively (Figure 1).

### 2.2. Biological Activity

**Figure 3:**
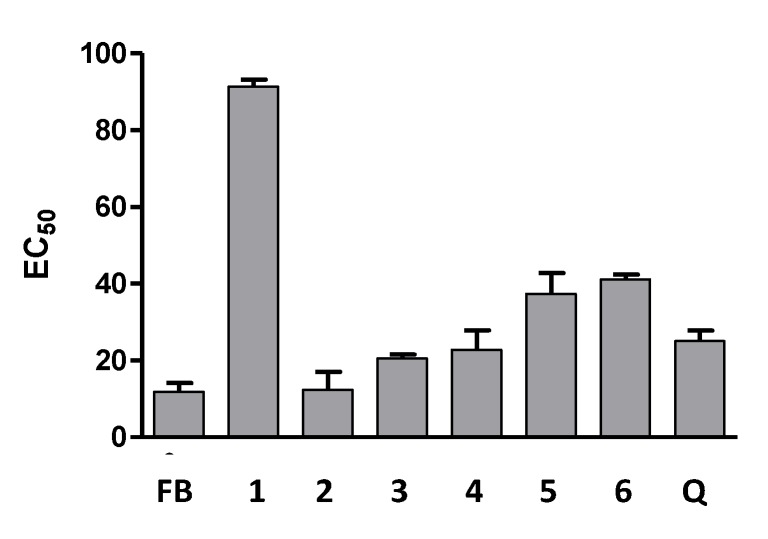
Antioxidant activity against LDL peroxidation of biflavonoids and **BF**. Results in μg/mL (mean ± S.D; n = 3).

All compounds and the BF were tested for their antioxidant activity against LDL-peroxidation (Figure 3). The flavanone-(3→8'')-flavone biflavonoids displayed antioxidant activity, particularly compound ** 2**, which was significantly more potent than quercetin, with a CE_50_ of 12.36 μg/mL (*p*-value < 0.05). The antioxidant potential of the BF was evaluated and correlated with its biflavonoid content, which was identified as amentoflavone (**1**), morelloflavone (**2**) and volkensiflavone (**3**). Cu^2+^-induced LDL oxidation was significantly reduced in the presence of the BF (EC_50_ = 11.85 μg/mL), mainly due to biflavonoid **2**, although synergy processes might also be involved. In fact, it has been reported that kolaviron, a biflavonoid fraction composed by GB-1, GB-B2 and kolaflavanone, increases lipoprotein resistance to copper-induced oxidation in rats, and also, * in vitro*, it protects against Cu^2+^-induced oxidation of rat serum lipoprotein, presumably by mechanisms involving metal chelation and antioxidant activity [29].

## 3. Experimental

### 3.1. General Experimental Procedures

^1^H, ^13^C-NMR, COSY, HMQC, and HMBC spectra were recorded on a Mercury 400F (400 MHz/100 MHz) spectrometer in CD_3_OD or DMSO-*d* (Agilent Technologies, Santa Clara, CA, USA). Optical rotations were carried out on a Perkin–Elmer 241 Polarimeter (Waltham, UK). UV spectra were recorded on a Hitachi U-2000 spectrophotometer (Hitachi High-Tech, Tokio, Japan). IR spectra were recorded on a Thermo Scientific Smart Miracle Spectrometer (Waltham, UK). HR-ESI-MS spectra were obtained on an LC/MSD-TOF (2006) mass spectrometer (Agilent Technologies, Santa Clara, CA, USA). A Jasco-J-810 spectropolarimeter (Jasco Incorporated, Easton, MD, USA) was used to run the CD spectra, all recorded in MeOH.

### 3.2. Plant Material

The aerial parts of *G. madruno* were collected in Medellín (Colombia). This sample was identified by M.Sc. Fernando Alzate. A voucher specimen (Alz-3030) has been deposited at the Herbarium of Universidad de Antioquia (HUA).

### 3.3. Extraction and Isolation

The dried material of *G. madruno* (1.8 kg) was successively extracted with hexane (3 × 8 L), EtOAc (3 × 8 L) and MeOH (3 × 8 L). After evaporation, a portion of the EtOAc extract (50 g) was fractionated by VLC on silica gel (6–35 μm; 8 × 10 cm) and eluted with a hexane, hexane-EtOAc, EtOAc-MeOH gradient solvent system to give eight main fractions (A-H) on the basis of their TLC (silica gel, hexane-EtOAc 8:2; methanolic ferric chloride) behaviour. Fraction C (7.72 g) was subjected to CC on silica gel (40–63 μm; 2 × 40 cm) using hexane-EtOAc (8:2) as the solvent to give five fractions (C 1–5). Crystallisation of combined fractions 1–3 with hexane-EtOAc (3:1) gave 1 (0.47 g); Fraction 4–5, called BF, was rechromatographed by preparative TLC, eluting with EtOAc–Ether–BuOH (6:3:1) to give 1 (10 mg; R_f_ 0.55), 2 (0.23 g; R_f_ 0.50), and 3 (15 mg; R_f_ 0.47). The MeOH extract was fractionated by dissolving it in hexane to give a soluble (71.5 g) and insoluble (48.2 g) fraction. The insoluble fraction was subjected to VLC and treated in the same manner as the EtOAc extract to give six main fractions labelled M 1-6. The pure compound 4 (0.6 g) was obtained by direct crystallisation with hexane–EtOAc (2:1) from fraction M-1. Combined fractions M 2–4 (5.36 g) were subjected to preparative TLC eluting with CH_2_Cl_2_–MeOH (9:1) to give more 4 (82 mg; R_f_ 0.22), 5 (6 mg; R_f_ 0.18) and 6 (8 mg; R_f_ 0.15).

### 3.4. 7''-O-(6''''-acetyl)Glucoside of Morelloflavone (**5**)

Brown powder; m.p. 268–270 °C; 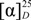 + 155 (c 0.10, MeOH); CD (5.0 × 10^−5^ M) 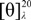: [θ]_288_ +67,394, [θ]_351_ +36,263 (MeOH); UV λ (nm): 332, 292, 209, MeOH_max_; IR (KBr) ν*_max_* cm^−1^: 3,168, 1,648, 1,536, 1,316; ^1^H-NMR and ^13^C-NMR see Table 1; HR ESIMS *m/z* 761.1720 [M+H]^+^ (calcd for C_38_H_32_O_17_, 761.1718).

### 3.5. Inhibition of LDL Oxidation

The protective effect of the biflavonoids and the BF against LDL-peroxidation was determined by a TBARS assay.

#### 3.5.1. Human LDL Isolation

50 mL of blood was collected by venepuncture into heparinized tubes from healthy non-smoking volunteers (20–25 years old). Plasma was recovered by differential density ultracentrifugation at 2,500 rpm and 4 °C, in a Beckman XL-100 ultracentrifuge (Brea, CA, USA) equipped with a SW-55Ti rotor, as described elsewhere. The LDL fraction was obtained by centrifugation with 1.6 mL of NaCl (17 M) in distilled water at 49,500 rpm for 12 h. The superior fraction was removed and 1.6 mL of KBr (10 M) was added before another centrifugation for a period of 18 h. SDS-PAGE was used to confirm the purity of the collected fractions (kilomicrons, VLDL, LDL and HDL). The concentration of protein was determined by the Protein Quantification Kit-Rapid method of Fluka^®^ (St. Louis, MO, USA).

#### 3.5.2. TBARS Determination

The formation of products from peroxidation of LDL was determined by the thiobarbituric acid reactive substances assay (TBARS). The LDL was incubated at 37 °C in 0.1 M potassium phosphate buffer, and made up to a final protein concentration of 300 μg/mL. Volumes of 50 μL of biflavonoids or quercetin (positive control) at different concentration were added and the peroxidation was initiated by 50 μL of CuSO_4_ 100 μM, and finished by 5 μL of 1% EDTA and cooling. A TCA–TBA–HCl stock solution (15% w/v trichloroacetic acid; 0.67% w/v thiobarbituric acid; 0.1N HCl) was added to the reaction mixture. The solution was then heated at 95 °C for 60 min. The supernatant was filtered through a 0.45 μm membrane and a reading was made at 532 nm. Readings of three independent experiments were carried out. One-way ANOVA with a Student Newman-Keuls post-test was performed using GraphPad Prism version 4.00 and *p* < 0.05 was considered as significant difference.

## 4. Conclusions

In summary, six biflavonoids were isolated from *G. madruno*. Compound **5** was found to be a new biflavonoid glycoside on the basis of spectroscopic analyses and chemical evidence. Some of the compounds exhibited potent lipid peroxidation inhibition activity, and lipid peroxidation was also significantly reduced in the presence of the biflavonoid fraction (BF), mainly due to morelloflavone. These results suggest that the BF is an excellent antioxidant.
